# Renal Cell Tumors: Uncovering the Biomarker Potential of ncRNAs

**DOI:** 10.3390/cancers12082214

**Published:** 2020-08-07

**Authors:** Gonçalo Outeiro-Pinho, Daniela Barros-Silva, Margareta P. Correia, Rui Henrique, Carmen Jerónimo

**Affiliations:** 1 Cancer Biology and Epigenetics Group, IPO Porto Research Center (CI-IPOP), Portuguese Oncology Institute of Porto (IPO Porto), Rua Dr. António Bernardino de Almeida, 4200-072 Porto, Portugal; goncalo.outeiro.pinho@ipoporto.min-saude.pt (G.O.-P.); daniela.silva@ipoporto.min-saude.pt (D.B.-S.); margareta.correia@ipoporto.min-saude.pt (M.P.C.); henrique@ipoporto.min-saude.pt (R.H.); 2 Department of Pathology, Portuguese Oncology Institute of Porto, 4200-072 Porto, Portugal; 3 Department of Pathology and Molecular Immunology, Institute of Biomedical Sciences Abel Salazar-University of Porto (ICBAS-UP), Rua de Jorge Viterbo Ferreira n. 228, 4050-313 Porto, Portugal

**Keywords:** Renal cell tumors, renal cell carcinoma, biomarkers, liquid biopsies, diagnosis, prognosis, non-coding RNAs, miRNA, lncRNA

## Abstract

Renal cell tumors (RCT) remain as one of the most common and lethal urological tumors worldwide. Discrimination between (1) benign and malignant disease, (2) indolent and aggressive tumors, and (3) patient responsiveness to a specific therapy is of major clinical importance, allowing for a more efficient patient management. Nonetheless, currently available tools provide limited information and novel strategies are needed. Over the years, a putative role of non-coding RNAs (ncRNAs) as disease biomarkers has gained relevance and is now one of the most prolific fields in biological sciences. Herein, we extensively sought the most significant reports on ncRNAs as potential RCTs’ diagnostic, prognostic, predictive, and monitoring biomarkers. We could conclude that ncRNAs, either alone or in combination with currently used clinical and pathological parameters, might represent key elements to improve patient management, potentiating the implementation of precision medicine. Nevertheless, most ncRNA biomarkers require large-scale validation studies, prior to clinical implementation.

## 1. Renal Cell Tumors

Renal cell tumors (RCT) rank 16th among the most common neoplasms in adults, representing more than 400,000 new cases yearly (2.2% of all cancer diagnosis) in both genders, with a mortality rate of 2.4/100,000, worldwide [1]. RCTs are a heterogenous group of tumors, spanning from benign to overtly malignant behavior and being highly diverse at the molecular, genomic/epigenomic, morphological, and clinical level [2]. Benign renal tumors correspond to 10–13% of all RCT, being oncocytomas the most prevalent, whereas clear cell renal cell carcinomas (ccRCC) are the most common and one of the most aggressive malignant RCT subtypes (70–75% of all cases), followed by papillary renal cell carcinomas (pRCC, 10–15%) and chromophobe renal cell carcinomas (chRCC, 5–10%) [3]. Since these four types of RCT represent the vast majority of renal tumors, they will represent the main focus of this review. Although, in recent years, mortality rate has dropped, incidence has increased, mainly due to incidental detection. Indeed, more than 50% of RCTs are incidentally detected after nonspecific musculoskeletal or gastrointestinal complaints entailing abdominal imaging [4]. Visible and/or palpable manifestations, such as flank pain, hematuria, and abdominal mass are infrequent, only observed in a small number of cases, and are mostly associated with advanced disease [5]. Thus, physical examination does not allow for early diagnosis of RCT. Presently, partial or radical nephrectomy is the main curative treatment available since these tumors are notably resistant to both radio- and chemotherapy [6]. However, cases of complete curative treatment have been reported with interleukin-2 (IL-2) and nivolumab-based therapy [7,8]. The clinical benefit of adjuvant interferon-alpha (IFN-α) and IL-2, heat shock protein-peptide complex-96 (HSPPC-96, Vitespen^®^), girentuximab, or vascular endothelial growth factor receptor/tyrosine kinase inhibitor (VEGFR/TKI) for high-risk RCT patients remains unclear, as results of published randomized trials are conflicting [9,10,11,12,13,14]. Furthermore, 30 to 35% of the cases are diagnosed with locally invasive or distant disease, and 20 to 40% of the patients without metastasis at the time of diagnosis will develop metastatic dissemination during the disease course [15]. For metastatic renal cell carcinoma (mRCC), VEGFR/TKI antiangiogenic drugs, such as pazopanib, sunitinib, or cabozantinib, have been shown to improve disease control [16,17,18]. In patients where antiangiogenic agents are inefficient, the use of mammalian target of rapamycin (mTOR) pathway inhibitors, such as everolimus and temsirolimus, has shown favorable results [19]. Lastly, a new wave of immunotherapy-based approach is arising and, nivolumab, a programmed cell death 1 (PD-1) blocking antibody, and atezolizumab, a programmed cell death-ligand 1 (PD-L1) blocking antibody, have also demonstrated promising results by increasing mRCC overall survival (OS) [20,21]. According to the American Cancer Society, patients with localized disease present a five-year survival rate above 75%, whereas for mRCC patients it decreases to less than 15%. Poor prognosis of advanced RCC can be explained by a wide variety of factors, with the acquired resistance to targeted therapies the main one [22]. Currently, no adequate tools for the screening or early diagnosis of RCT are available. Furthermore, prognostication is mainly based on clinical stage and metastatic dissemination, and therapy efficacy is rather poor. Thus, the development and clinical implementation of more robust, reliable, and cost-effective biomarkers capable of RCTs’ early-stage detection and/or prediction of disease progression and therapy response is mandatory. To tackle these limitations, tumor-related genetic and/or epigenetic alterations may be used as biomarkers [23], ultimately improving patient survival and quality of life, while reducing healthcare costs through avoidance of futile therapeutic interventions.

## 2. Epigenetics

**Figure 1:**
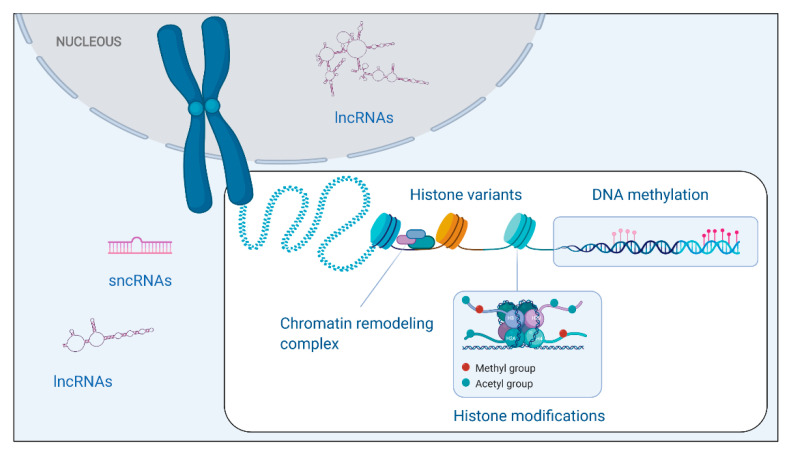
Schematic representation of epigenetic machinery components. DNA methylation is catalyzed by DNA methyl transferases (DNMT) and consists of covalent addition of a methyl group to the 5-carbon of the cytosine ring resulting in 5-methylcytosine (5-mC). Histone post-transcriptional modifications constitute another type of epigenetic-based gene regulatory mechanism. These reactions (e.g., acetylation and methylation) occur in the residues of histone tails and are extremely refined, being catalyzed by highly regulated enzymes. In addition, histones might also have variants, which alter nucleosome functionality. Chromatin remodeling complexes, such as SWItch/Sucrose Non-Fermentable (SWI/SNF), Imitation SWI (ISWI), INO80, and Nucleosome Remodeling Deacetylase (NuRD), alter chromatin architecture, through direct interaction with nucleosomes. Finally, non-coding RNAs, namely small non-coding RNAs (sncRNAs) and long non-coding RNAs (lncRNAs) post-transcriptionally regulate gene expression, both in the nucleus and cytoplasm. Created by BioRender.com (https://biorender.com/)

Epigenetics, firstly termed by Conrad Waddington in 1942, refers to mitotically and/or meiotically heritable and reversible changes in gene expression, which do not alter primary nucleotide sequence [24]. Epigenetic regulation involves four major types of modifications: DNA methylation, histone modifications/variants, chromatin remodeling complexes, and non-coding RNAs (ncRNAs) [23,24]. The first three control chromatin architecture, regulating gene expression (Figure 1). The transcriptional outcome of DNA methylation is genome location-dependent, since gene promoter DNA methylation leads to transcription repression, while gene body DNA methylation is associated with transcription activation. Histone-tail methylation is residue-specific, often leading to repressive marks and increased chromatin condensation, while acetylation results in activation marks and looser chromatin architecture [23]. Abnormalities in the normal function of the epigenetic machinery have been linked to several human conditions, including cancer [23]. Epigenetic deregulation often occurs early in tumorigenesis leading to a switch in the normal epigenetic patterns and accumulates during disease progression [25]. It is acknowledged that some of these epigenetic alterations might occur prior to the emergence of the malignant phenotype, thus constituting a valuable marker for cancer screening. From a technical point of view, methodologies available to detect those epigenetic marks are sensitive and robust, enabling easy measurement across individuals and with high-throughput screening potential [26].

Global increase in RCT incidence over the last decades and the concerns regarding the most suitable follow-up and treatment for each patient demand reliable biomarkers amenable to clinical use. Herein, we aimed to critically review and highlight the most scientifically relevant and clinically promising studies concerning ncRNA-based biomarkers for RCT detection, prognostication, prediction of response to therapy, and patient monitoring.

## 3. Evidence Acquisition

Bibliography was selected after a PubMed search up to 19 April 2020 using the keywords: Non-coding RNA, biomarkers, and renal cell tumor, which resulted in the analysis of more than 400 manuscripts. All articles’ references were also examined for potentially useful studies. Furthermore, relevant articles were selected based on the following criteria: Written in English, the main topic is non-coding RNA, biomarkers, and renal cell carcinoma. Original reports were chosen based on the detail of analysis, mechanistic support of data, novelty, and potential clinical usefulness of the findings. After thorough analysis, 143 original articles were enrolled in the final version of this review.

## 4. Non-Coding RNAs (ncRNAs)

Although most of the genome is transcribed into RNAs, only a small percentage encodes for proteins (1–2%). Thus, most RNAs are, indeed, ncRNAs, devoid of protein-coding potential [27]. For many years, ncRNAs were thought to be “transcriptional trash”. However, this perception has recently changed, and the pivotal roles of ncRNAs in major biological processes, such as imprinting, cell cycle, pluripotency, and gene expression regulation, are now widely acknowledged [28,29,30]. Based on the functional RNA molecule’s size, ncRNAs are further categorized into small non-coding RNAs (sncRNAs) if smaller than 200 base pairs in length [31,32] or long non-coding RNAs (lncRNAs) [33,34].

### 4.1. Small Non-Coding RNAs (sncRNAs)

The classification of sncRNAs as epigenetic mechanism of gene expression control remains controversial. Several studies have pointed that sncRNAs’ mechanism of action is post-transcriptional and should not be thus classified as epigenetic regulators, whereas others have a contrasting view. Nevertheless, this subclass of ncRNAs is biologically relevant. MicroRNAs (miRNAs) are the most well studied of these small molecules [35]. This class of small ncRNAs are 18-25 nucleotides in length [24] and regulate gene expression through RNA interference (RNAi) [23]. In the human genome, miRNAs are encoded by individual genes or clusters of few to several hundred different miRNAs genes [36]. The latter are then transcribed as polycistronic transcripts, which are ultimately processed into the individual mature miRNAs. In most cases, miRNAs are encoded by introns of non-coding or coding genes, but they can also be encoded by exonic regions [37]. Following transcription by RNA polymerase II, the primary miRNA (pri-miRNA) undergoes several steps of maturation, catalyzed by type III ribonucleases (RNases). First, in the nucleus, the Drosha complex cleaves the pri-miRNA, leading to the formation of the precursor miRNA (pre-miRNA). Then, after pre-miRNA transport to the cytoplasm, Dicer complex cleaves the molecule, generating a miRNA duplex, which is loaded into the pre-miRNA-inducing silencing complex (pre-miRISC), where the stable 5′ end strand—guide strand—is selected, generating the mature miRISC complex, whereas the other strand—passenger strand—is rapidly degraded [38]. Together with GW182 family of proteins, miRISC binds to mRNA targets by base complementarity and, ultimately, leads to gene silencing. Another type of sncRNAs are the P-element Induced Wimpy testis (PIWI)-interacting RNAs (piRNAs). First discovered in the beginning of the 21st century [39], these 21–35 nucleotide-long molecules are involved in viral infection response, transposable elements silencing, and regulation of gene expression by (1) leading PIWI proteins to cleave target RNA, (2) promoting heterochromatin assembly, and (3) inducing DNA methylation [40,41]. One of the main features that distinguishes miRNAs from piRNAs is that the latter have single-strain RNA precursors and its processing requires PIWI proteins of the Argonaute/PIWI family, but does not need DICER complex [42]. Small interfering RNAs (SiRNAs) are also classified as sncRNAs [31], but this review solely focused on miRNAs and piRNAs, as these are the most well studied.

#### 4.1.1. MiRNAs and piRNAs in RCTs

Deregulated miRNA expression in cancer was first reported in the early 2000s by Calin and colleagues [43]. Since then, various studies have demonstrated differential miRNA expression profiles in benign and malignant neoplasms compared to healthy individuals. Dysregulation of miRNA expression occurs in various steps of tumorigenesis and in several tumor models [23], including RCT [24,44]. MiRNAs possess the ability to target several mRNAs, and one mRNA might be targeted by many miRNAs [45]. Depending on the target, miRNAs are classified as tumor suppressor miRNAs (TSmiRs) or as oncogenic miRNAs (oncomiRs). TSmiRs are usually downregulated in cancer and act through transcriptional repression of oncogenes, whereas oncomiRs are normally upregulated, and act by targeting tumor suppressor genes, leading to mRNA decay and/or degradation [46]. Nonetheless, several reports have demonstrated that, depending on the cellular context and the tumor type, the same miRNA may exhibit oncogenic or tumor suppressor activity, such as let-7g, which is downregulated in lung cancer and upregulated in colorectal cancer [47,48].

Concerning piRNAs, most are not complementary to putative target mRNAs, indicating that piRNAs may be involved in epigenetic regulation rather than post-transcriptional regulation, controlling a variety of biological processes and being also implicated in cancer development [49]. Several studies aimed to disclose their biological role in different cancer types [50], including RCC [51,52,53]. However, the specific molecular mechanism underlying piRNAs’ deregulation in carcinogenesis is still poorly understood, and further investigation is needed.

Due to their tissue and cellular-specific functions and expression, the potential use of sncRNAs as diagnostic, prognostic, predictive, and monitoring cancer biomarkers has been extensively studied in the recent years. Here, we highlighted the most promising findings in RCT, both in tissue and liquid biopsies.

#### 4.1.2. SncRNAs as Diagnostic Biomarkers

##### Tissue-Based Samples

The increasing number of asymptomatic, incidentally detected renal masses constitutes a major clinical challenge, considering the need to define the potential threat to the life of the patient. Whether a biopsy is mandatory or not remains controversial, considering that histopathological and/or cytopathological assessment may not provide a definitive diagnosis in a sizeable proportion of cases. Thus, the ability of sncRNAs to discriminate between normal and benign/malignant tissue has been investigated. Wotschofsky and co-workers [54] measured the differential expression of several miRNAs in a series of 111 ccRCC and matched normal tissue (MNT) using quantitative real-time PCR (RT-qPCR). The combination of miR-141, miR-155, and miR-184 identified malignancy with 95% sensitivity, 100% specificity, corresponding to an area under curve (AUC) of 0.990 [54]. In another study, miR-141 or miR-200b levels discriminated RCC from normal renal tissue (NRT) with 99.2% sensitivity, 100% specificity, and an AUC of 0.991. Furthermore, the same panel distinguished ccRCC, pRCC, or chRCC from benign renal tumors with 85.6% sensitivity, 100% specificity, and an AUC of 0.914 [55]. In 2015, Busch and colleagues [51] reported that piR-30924, piR-57125, and piR-38756 were differentially expressed in ccRCC compared to NRT and the combination of these piRNAs identified malignant disease with 91% sensitivity, 86% specificity, and an AUC of 0.910. Notably, the combination of the duo piR-30924 and piR-57125 distinguished metastatic-ccRCC (mccRCC) from non-metastatic ccRCC (non-mccRCC) with 73.0 sensitivity, 74.0 specificity, and an AUC of 0.760 [51]. Several other studies have been published since, and are summarized in Table 1. Because tissue biopsies are seldom performed and might not represent the entire lesion, these biomarkers might assist in the correct classification of the tumor. Moreover, this is an invasive procedure, which submits patients to stress and pain, eventually associated with increased risk of metastization, especially in ccRCC. Hence, discovery and validation of non-invasive screening/diagnosis biomarkers, capable of accurately identifying the nature of renal masses, is urgently needed.

##### Liquid Biopsies

Recently, detection and characterization of circulating sncRNAs might represent a promising non-invasive technique to identify RCT [76]. SncRNAs are highly stable and abundant in plasma, serum, and other body fluids, being released from damaged or apoptotic normal cells, as well as from tumor cells. Numerous reports have proposed several RCT biomarkers in liquid biopsies. Serum samples were firstly used in a study by Wulfken and colleagues [77], which demonstrated that serum miR-1233 expression levels discriminated cancer patients from asymptomatic controls (AC) with 77.4% sensitivity and 37.6% specificity. The limited performance of this marker compared to tissue-based studies might be explained by technical limitations. Since then, methodology has improved, and miR-210 expression levels were found to discriminate ccRCC and AC in serum samples with 81.0% sensitivity and 79.4% specificity [78]. Recently, miR-1233 and miR-210 levels, in serum and in exosomes, discriminated ccRCC from healthy controls with 81.0/70.0% sensitivity and 76.0/62.2% specificity, respectively, with exosome-derived samples showing a better biomarker performance [79]. Moreover, plasma samples have also been tested. Specifically, miR-21 and miR-106a isolated from plasma (30 ccRCC and 30 AC) disclosed the ability to identify renal malignancy with 77.3% sensitivity and 96.4% specificity for the former miR and 86.7% sensitivity and 70.0% specificity for the latter [80]. Subsequently, Lou and colleagues [81] showed that miR-144-3p detected RCT with 87.1% sensitivity and 83.0% specificity. Notably, miR-144-3p was also able to distinguish ccRCC from benign mesenchymal tumors (angiomyolipomas) with 75.0% sensitivity and 71.7% specificity [81]. Finally, diagnostic biomarkers have also been tested in urine samples. In 2016, Butz and colleagues [82] reported that miR-126-3p and miR-34b-5p, isolated from urine exosomes, could discriminate ccRCC from healthy controls with 77.5% sensitivity and 72.4% specificity. Remarkably, both miRs also distinguished benign lesions from normal with 75.0% sensitivity and 82.8% [82]. Additionally, urinary miR-15a expression levels, evaluated in 67 RCT patients and 15 AC, detected malignancy with 98.1% sensitivity and 100% specificity [83]. A summary of these and other studies is depicted in Table 2.

#### 4.1.3. SncRNAs as Prognostic Biomarkers

##### Tissue-Based Samples

Several sncRNAs have also been proposed as predictors of disease progression and outcome. Currently, RCT prognosis is mainly based on clinical stage and other clinical parameters at diagnosis. Nonetheless, specific sncRNAs might complement the currently used clinicopathological parameters, to improve patient management. In 2013, Wang and colleagues [96] reported that RCC patients disclosing higher miR-100 expression levels endured significantly shorter overall survival (OS), multiplying by a factor of three the risk of death comparing to those with low expression. Likewise, increased miR-630 expression levels independently predicted shorter OS, in multivariable analysis [97]. Importantly, sncRNAs have shown promise as predictors of disease-progression. Samaan and colleagues [98] divided their 258 ccRCC patient cohort into either miR-210 positive or negative expression groups. The first group of patients displayed markedly reduced disease-free survival (DFS) (hazard ratio (HR): 1.91; 95% confidence interval (CI): 1.10–3.310) compared to the negative expression group [98]. The same trend was observed in two subsequent studies, in which higher miR-210 expression associated with worse survival [99,100], whereas in another study increased miR-210 expression levels in ccRCC tissue associated with better survival [101]. Thus, multicentric studies with larger cohorts are needed to unveil the exact prognostic value of miR-210. Furthermore, high miR-27a-3p expression levels associated with shorter progression-free survival (PFS) [102], whereas low miR-155 expression entailed 5-fold increase risk to die from the disease. Notably, both miR-27a-3p and miR-155 expression levels were independent predictors of cancer-specific survival (CSS) in advanced ccRCC (stages III and IV) [103]. Table 3 summarizes these and other relevant findings concerning the prognostic value of miRNAs in RCC.

##### Liquid Biopsies

Studies on sncRNAs as potential biomarkers for RCC progression and/or disease outcome in liquid biopsies are rather scarce. Let–7i–5p low expression in exosomes from plasma of 65 mRCC patients associated with shorter OS [133]. Fujii and colleagues [134] showed that higher plasma-derived exosomal miR–224 expression levels negatively associated with shorter OS, CSS, and recurrence-free survival (RFS). A subsequent analysis of 67 ccRCC serum samples demonstrated that increased miR–206 and miR–122–5p expression associated with increased risk of disease progression and mortality, although, in multivariable analysis, only miR–206 retained independent value as predictor of PFS [135]. Finally, Dias and colleagues reported that higher miR–210, miR–221, and miR–1233 plasma levels associated with shorter CSS [99]. Detailed information of all relevant studies may be found in Table 4.

#### 4.1.4. SncRNAs as Predictive Biomarkers of Response to Therapy

##### Tissue-Based Samples

Uncertainties concerning efficacy and deleterious side effects of RCC therapy negatively impact patient management [136,137,138]. Ideally, each patient should be prescribed the therapy most likely to specifically target and eliminate neoplastic cells, which sets the basis for precision medicine [139]. Considering their involvement in critical metabolic pathways, it is unsurprising that sncRNAs have been implicated in cancer therapy resistance [140,141,142]. Furthermore, sncRNAs have been proposed as predictors of response to therapy in RCC. Indeed, miR–141 expression levels were shown to predict response to sunitinib, as patients with low levels disclosed a significantly worse response [143]. In a different study, lower expression levels of both miR–155 (a well-known oncomiR) and miR–484 (with biological role yet to be fully understood) associated with increased time to progression (TTP) in a series of 63 mRCC patients (44 responders and 19 non-responders) treated with sunitinib [144]. Recently, Go and colleagues demonstrated that miR–421 was highly expressed in RCC tissues from patients who did not respond to VEGFR–TKI [145]. Table 5 provides additional information on the most relevant studies concerning the predictive value of sncRNAs in RCC.

##### Liquid Biopsies

One of the main drawbacks of tissue-based studies is the inability to capture the dynamic nature of sncRNAs’ expression along time, either during disease progression or due to therapeutic intervention. The usage of liquid biopsies might circumvent this limitation, since it allows sample collection at several time points, i.e., prior to, during, and after treatment, enabling patient monitoring. In 2012, Gámez–Pozo and colleagues analyzed the expression of 287 miRs in 38 whole blood samples from patients with advanced RCC treated with sunitinib and constructed multiple models of poor and prolonged response to this TKI. Notably, miR–410, miR–1181, and miR–424 downregulation was associated with prolonged response, whereas low miR–192, miR–193a–3p, and miR–501–3p levels associated with limited response [146]. Additionally, serum miR–605 levels in mccRCC patients treated with vorinostat and bevacizumab were exclusively reduced in the responders’ group, comparing to the disease progression group [156]. Additional studies are summarized in Table 5.

##### In Vitro Studies

Several studies have been performed in RCC in vitro models in the pursuit of both predictive biomarkers and insights on therapy-resistance mechanisms. Although in vitro models do not fully mimic biological conditions, they allow for the discovery of potential sncRNA-based biomarkers, which may be subsequently validated in clinical samples. Gao and colleagues [149] reported that transfecting mimic miR–200c into ccRCC cell lines resistant to imatinib and sorafenib re-sensitized cells to therapy. Moreover, miR–200c was downregulated in RCC cell lines, whereas one of its targets, CYP1B1, was overexpressed. Remarkably, increased CYP1B1 levels were associated with docetaxel resistance [152]. Additionally, miR–101 downregulation in ccRCC cell lines associated with high UHRF1 (a miR–101 target) levels and, ultimately, to sunitinib resistance [163]. Detailed information on these and other studies can be found in Table 5.

### 4.2. LncRNAs

As previously mentioned, lncRNAs are a class of ncRNAs with more than 200 base pairs in length [33,34]. These molecules can be further categorized into different groups, depending on their genome location, sequence, morphology, structure, and functional features [164,165]. Most lncRNAs are synthesized by the same biogenesis machinery as mRNAs and endure post-transcriptional modifications, such as 5′ terminal methylguanosine cap (5′ cap), are often spliced in a canonical manner, and some are 3′ polyadenylated [165]. LncRNAs have a fine-tuned regulation by transcription factors and typically display a tissue-specific expression profile [164]. Functionally, lncRNAs have been implicated in a multitude of biological processes, such as nuclear organization through nucleosome remodeling [166], gene-to-gene interactions [167], and as regulators of miRNA expression [168], thus prompting the hypothesis that lncRNAs’ differential expression might be associated with human disease. Indeed, several pathological conditions display aberrant lncRNAs’ expression profile [169], including cancer [170].

#### 4.2.1. LncRNAs in RCT

LncRNAs have been the focus of several research studies aiming at the discovery of novel biomarkers and understanding the biological mechanisms through which they influence the genesis and progression of RCT [171,172,173]. Compared to their protein-coding counterparts, lncRNAs are considerably less expressed, which might constitute a major pitfall for their use in clinical practice, since robust detection is quite challenging [174]. Nonetheless, the study of these molecules should be promoted, as technological advances might overcome the present limitations. Herein, we highlighted the most relevant studies reporting lncRNAs as potential diagnostic, prognostic, predictive, and monitoring biomarkers in RCT, both in tissue and liquid biopsies.

#### 4.2.2. LncRNAs as Diagnostic Biomarkers

##### Tissue-Based Samples

Contrarily to sncRNAs, published data concerning lncRNAs as RCT diagnostic biomarkers are limited. Over two decades ago, Thrash–Bingham and colleagues [175] reported, for the first time, that lncRNA expression was dissimilar among RCC subtypes. Using semiquantitative PCR, a marked increased expression of lncRNA antisense Hypoxia Inducible Factor (aHIF) in ccRCC, comparatively to pRCC, was disclosed [175]. Technology has evolved and these results were subsequently validated in 2011, when Bertozzi and colleagues [176] detected a differential expression of lncRNA aHIF between RCC and MNT, as well as between non-pRCC and pRCC tissue samples. In another study, comprising 102 ccRCC and 50 NRT, lncRNA CYP4A22–2/3 discriminated ccRCC from NRT with an AUC of 0.790 [177]. In 2016, the expression of lncRNA UC009YBY.1 and lncRNA ENST00000514034 was assessed in a set of 70 ccRCC and 70 MNT by Ren and colleagues [178]. These authors reported that the two lncRNAs could identify RCC tissue with 54.29% sensitivity and 82.86% specificity for the former, and 60.00% sensitivity and 67.14% specificity for the latter [178]. Finally, a recent study reported that lncRNA HOX Transcript Antisense RNA (HOTAIR) might also constitute a ccRCC diagnostic biomarker, disclosing an AUC of 0.9230 [71]. Table 6 summarizes the complete information on relevant published studies reporting lncRNAs as potential RCC diagnostic biomarkers.

##### Liquid Biopsies

Based on our literature search, only two relevant studies evaluating the potential of lncRNAs as RCC diagnostic biomarkers in liquid biopsies were found. Wu and colleagues [183] assessed the expression of five lncRNAs (lncRNA–low expression in tumor (LET), Plasmacytoma Variant Translocation 1 (PVT1), Promoter Of CDKN1A Antisense DNA Damage Activated RNA (PANDAR), Phosphatase and Tensin Homolog Pseudogene 1 (PTENP1), and long intergenic non-protein coding RNA 963 (linc00963)) in two sets of ccRCC and AC serum samples. When combined in a panel, these biomarkers identified malignancy with 79.2% sensitivity and 88.9% specificity in the training set (24 ccRCC and 27 AC) and with 67.6% sensitivity and 91.4% specificity in the testing set (37 ccRCC and 35 AC) [183]. Subsequently, serum expression of lncRNA GIHCG was assessed in a set of 46 ccRCC and 46 AC samples. GIHCG expression discriminated ccRCC from healthy donors with 87.0% sensitivity and 84.8% specificity. Remarkably, it could also distinguish early-stage ccRCC from AC (31 stage I ccRCC vs. 46 ACs) with 80.7% sensitivity and 84.8% specificity [184]. The complete information concerning these studies is provided in Table 6, together with tissue-based studies.

#### 4.2.3. LncRNAs as Prognostic Biomarkers

##### Tissue-Based Samples

As for sncRNAs, several reports on the potential of lncRNAs as RCC prognostic biomarkers in tissue samples have been published. In a series of 102 ccRCC, Ellinger and colleagues [177] showed that patients disclosing low lncRNA Zinc-Finger protein 180-2 (ZNF180-2) expression endured significantly shorter OS. Notably, lower expression of this lncRNA also correlated with shorter CSS and PFS [177]. In another study, expression levels of lncRNA regulator of Akt Signaling Associated With HCC And RCC (lncARSR) were assessed in a set of 205 ccRCC tissues from patients subdivided into high- and low-expression groups. Patients with lncARSR–high expression displayed significantly shorter RFS, doubling the risk of recurrence comparatively to the lncARSR–low expression group [185]. Furthermore, Bao and colleagues [186] reported that lncRNA PVT1 could serve as independent prognostic biomarker in ccRCC. Indeed, patients with high lncRNA PVT1 expression depicted 1.5 and 3.5 times higher risk of death or recurrence, respectively, compared to patients with low lncRNA PVT1 expression [186]. Recently, in a series of tissues from 204 ccRCC patients, Wang and colleagues [187] found that high lncRNA EGFR-antisense RNA 1 (EGFR–AS1) expression levels increased two-fold the risk of death. Table 7 depicts the complete information on these and other relevant studies assessing the potential of lncRNAs for RCC prognostication.

##### Liquid Biopsies

After literature revision, only two studies reporting on the potential of lncRNAs for RCC’s prognostication in liquid biopsies were found. Qu and colleagues [204] assessed the expression of lncARSR in 71 ccRCC plasma samples and verified that higher expression levels were associated with worse PFS. In the other study, serum samples from 46 ccRCC patients were analyzed and increased GIHCG expression levels associated with lower OS [184]. However, both studies lack biomarker prognostic performance analysis, preventing a more robust evaluation of the potential of lncRNAs assessed in liquid biopsies. Table 7 provides the complete information on these two studies.

#### 4.2.4. LncRNAs as Predictive Biomarkers of Response to Therapy

Considering the limited number of studies assessing the ability of lncRNAs to predict response to therapy in RCC, we decided not to subdivide this section according to sample source. Qu and colleagues analyzed 71 plasma samples from ccRCC patients treated with sunitinib, in which high lncARSR expression levels were found in patients with progressive disease and poor response [204]. In another study, lncRNA Sorafenib Resistance in Renal Cell Carcinoma Associated (lncSRLR) expression was determined in tissue samples from 53 ccRCC patients treated with sorafenib. Patients with poor or no response to sorafenib therapy showed significantly higher expression levels of that lncRNA, which correlated with shorter PFS. In vitro studies revealed that lncSRLR knockdown in sorafenib-resistant RCC cell lines resulted in increased sensitivity to the treatment [205]. Furthermore, studies on RCC cell lines demonstrated a potential positive feedback loop between sunitinib and lncRNA Suppressing Androgen Receptor in Renal Cell Carcinoma (SARCC). Upon treatment with this TKI, lncRNA SARCC expression levels increased, leading to decreased resistance to sunitinib therapy [206]. Finally, a recent study reported that patients with poor response to sorafenib therapy displayed lower lncRNA Growth arrest-specific 5 (GAS5) expression levels. In vitro and in vivo experiments disclosed that overexpression of lncRNA GAS5 resulted in increased sensitivity to sorafenib [207]. Complete information on these studies is summarized in Table 8.

## 5. Conclusions

**Figure 2:**
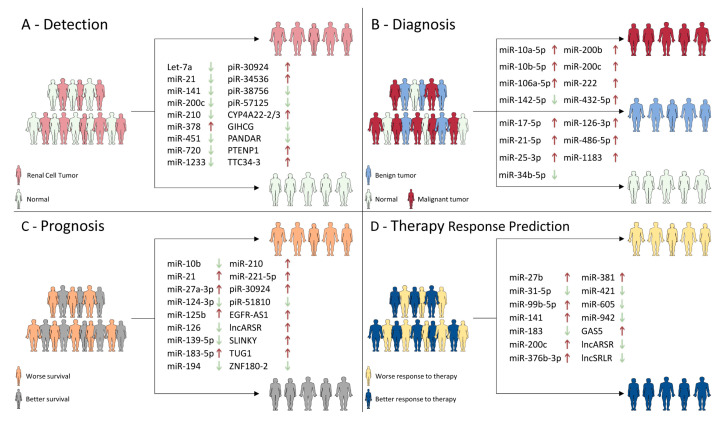
Summary of the most promising biomarker candidates for RCT. (**A**) Several ncRNAs have been proposed as ancillary tools for distinguishing tumor growth from normal tissue. Arrows represent each ncRNA expression level in RCT, comparing to normal samples (“red”—upregulated; “green”—downregulated). (**B**) Besides the potential use of ncRNAs to detect tumorigenesis, some were reported as capable to differentiate both malignant from benign lesions, and benign from asymptomatic conditions. The arrows represent ncRNA expression level (upper) in RCC, comparing to RCT and (lower) renal benign tumors, comparing to normal samples (“red”—upregulated; “green”—downregulated). (**C**) The potential of ncRNAs to stratify high-risk patients has been highly studied in recent years. The arrows represent each ncRNA expression level in patients displaying worse survival, comparing to those with better survival (“red”—upregulated; “green”—downregulated). (**D**) Lastly, ncRNA expression level has been proposed as potential predictor of therapy response. The arrows show the biological status of each ncRNA for a better response to therapy (“red’—upregulated; “green”—downregulated).

In summary, the data presented herein clearly support the feasibility of using ncRNAs as RCT biomarkers, both in tissue and liquid biopsies. Importantly, liquid biopsy-based samples are easily collected, minimally invasive, and may help to overcome the limitations of tissue biopsies. Further studies on ncRNAs should focus on technical and clinical validation, preferably using large-scale multicenter cohorts, allowing to determine whether these novel biomarkers may improve the clinical management of RCT patients. Finally, Figure 2 summarizes and illustrates the most promising ncRNAs for RCTs’ detection, diagnosis, prognostication, and prediction to therapy response included in this review.

## Figures and Tables

**Table 1:** Summary of proposed diagnostic biomarkers for Renal Cell Tumors (RCT) in tissue.

Year	Diagnostic Biomarker	Biological Source	Number of Cases/Controls	Diagnostic Performance			Reference
				Sensitivity (%)	Specificity (%)	AUC	
2009	miR-200c	Tissue	72 ccRCC; 72 MNT	n.a.	n.a.	0.970	[56]
2010	miR-200c	Tissue	13 chRCC; 21 oncocytomas	n.a.	n.a.	0.880	[57]
2012	miR-21	Tissue	71 ccRCC & 18 pRCC; 10 chRCC & 8 oncocytomas	83.0	90.0	0.886	[58]
2013	3 miR panel	Tissue	111 ccRCC; 111 MNT	95.0	100.0	0.990	[54]
2013	miR-210 + let-7c	Tissue	16 pRCC type I; 17 pRCC type II	n.a.	n.a.	0.919	[59]
2013	miR-200b	Tissue	90 RCC; 30 oncocytomas	96.7	90.0	0.914	[55]
2014	miR-3687	Tissue	24 ccRCC; 40 NRT	n.a.	n.a.	0.847	[60]
2014	miR-141	Tissue	68 ccRCC; 68 MNT	86.8	97.1	0.930	[61]
2014	miR-129-3p	Tissue	69 ccRCC; 69 MNT	75.9	62.1	0.735	[62]
2014	5 miR panel	Tissue	32 ccRCC; 16 NRT	100.0	100.0	1.000	[63]
2015	3 piRNA panel	Tissue	106 ccRCC; 77 NRT	91.0	86.0	0.910	[51]
2016	miR-145	Tissue	44 RCC; 44 MNT	n.a.	n.a.	0.616	[64]
2016	miR-141	Tissue	27 ccRCC; 27 MNT	n.a.	n.a.	0.912	[65]
2016	piR-823	Tissue	153 RCC; 121 MNT	n.a.	n.a.	0.795	[53]
2017	4 miR panel	Tissue	48 ccRCC; 50 benign renal tumors	91.7	94.0	0.992	[66]
2017	miR-34a	Tissue	85 RCC; 85 MNT	n.a.	n.a.	0.854	[67]
2017	miR-200c	Tissue	19 chRCC; 11 oncocytomas	84.0	82.0	0.820	[68]
2017	miR-200c	Tissue	30 ccRCC; 30 MNT	n.a.	n.a.	0.860	[69]
2017	miR-720	Tissue	30 RCC; 30 NRT	80.0	100.0	0.905	[70]
2018	miR-203	Tissue	53 ccRCC; 53 MNT	n.a.	n.a.	0.944	[71]
2018	miR-182-5p	Tissue	24 ccRCC; 24 MNT	n.a.	n.a.	0.954	[72]
2018	miR-224/miR-141	Tissue	68 ccRCC; 68 MNT	97.1	98.5	0.990	[73]
2018	miR-452-5p	Tissue	20 RCC; 20 MNT	n.a.	n.a.	0.919	[74]
2019	piR-34536	Tissue	118 ccRCC; 75 NRT	78.0	78.1	0.815	[75]

MNT—matched normal tissue; NRT—normal renal tissue; RCC—renal cell carcinoma; ccRCC—clear cell renal cell carcinoma; pRCC—papillary renal cell carcinoma; chRCC—chromophobe renal cell carcinoma; n.a.—not available.

**Table 2:** Overview of different proposed diagnostic biomarkers for RCT in liquid biopsies.

Year	Diagnostic Biomarker	Biological Source	Number of Cases/Controls	Diagnostic Performance			Reference
				Sensitivity (%)	Specificity (%)	AUC	
2011	miR-1233	Serum	84 RCC; 93 AC	77.4	37.6	0.588	[77]
2012	miR-378 + miR-451	Serum	90 RCC; 35 AC	81.0	83.0	0.860	[84]
2013	miR-210	Serum	68 ccRCC; 42 AC	81.0	79.4	0.874	[78]
2014	miR-210	Serum	34 ccRCC; 23 AC	65.0	83.0	0.770	[85]
2014	miR-221	Plasma	43 RCC; 34 AC	72.5	33.3	0.696	[86]
2015	5 miR panel	Serum	76 stage I ccRCC; 107 AC	80.0	71.0	0.807	[87]
2015	miR-210 + miR-378	Serum	195 RCC; 100 AC	80.0	78.0	0.848	[88]
2016	miR-126-3p + miR-486-5p	Urine exosomes	24 benign renal tumors; 33 AC	75.0	87.5	0.850	[82]
2016	piR-823	Serum	178 RCC; 101 AC	n.a.	n.a.	0.626	[53]
		Urine	20 RCC; 15 AC	n.a.	n.a.	0.743	
2017	miR-144-3p	Plasma	106 ccRCC; 123 AC	87.1	83.0	0.910	[81]
2017	miR-21	Plasma	30 ccRCC; 30 AC	77.3	96.4	0.865	[80]
2017	miR-210	Urine	75 ccRCC; 45 AC	57.8	80.0	0.760	[89]
2017	Let-7a	Urine	69 ccRCC; 36 AC	71.0	81.0	0.831	[90]
2017	miR-451	Plasma	94 ccRCC; 100 AC	n.a.	n.a.	0.640	[91]
2018	miR-1233	Serum exosomes	80 ccRCC; 82 AC	81.0	76.0	0.820	[79]
2018	miR-15a	Urine	67 RCT; 15 AC	98.1	100	0.955	[83]
2018	miR-210 × miR-224	Plasma	66 ccRCC; 67 AC	92.5	45.5	0.659	[73]
2018	miR-210	Serum exosomes	45 ccRCC; 30 AC	82.5	80.0	0.878	[92]
2019	miR-508-3p & miR-885-5p	Serum	85 ccRCC; 35 AC	n.a.	n.a.	0.900	[93]
2020	miR-432-5p	Urine	44 ccRCC-SRM; 27 oncocytomas	n.a.	n.a.	0.710	[94]
2020	miR-30a-5p^me^	Urine	171 ccRCC; 85 AC	63.0	67.0	0.670	[95]

RCC—renal cell carcinoma; ccRCC—clear cell renal cell carcinoma; RCT—renal cell tumor; ccRCC-SRM—clear cell renal cell carcinoma-small renal mass; AC—asymptomatic controls; me—promoter methylation; n.a.—not available.

**Table 3:** List of potential prognostic Renal Cell Carcinoma (RCC) biomarkers in tissue samples.

Year	Prognostic Variable	Prognostic Biomarker	Biological Source	Number of Cases	Poor Prognosis	Prognostic Performance		Reference
						HR	95% CI	
2010	RFS	miR-9-3	Tissue	59 ccRCC	High methylation	5.850	1.300–26.35	[104]
2012	CSS	4 miR panel	Tissue	68 ccRCC	High risk	8.800 *	2.620–29.58 *	[105]
2012	DFS	miR-21	Tissue	87 RCC	Positive expression	2.150 *	1.160–3.980 *	[58]
2013	RFS	miR-124-3	Tissue	80 ccRCC	High methylation	9.370	2.680–32.80	[106]
2013	RFS	miR-514	Tissue	87 ccRCC	Low expression	0.250	0.080–0.750	[54]
2013	OS	miR-210	Tissue	46 ccRCC	Low expression	3.010	1.390–6.510	[101]
2013	CSS	miR-486	Tissue	46 RCC	High expression	4.330	1.450–18.71	[107]
2013	OS	miR–100	Tissue	96 RCC	High expression	3.600	1.800–5.200	[96]
2013	CSS	miR–155	Tissue	137 ccRCC	Low expression	5.490	2.400–12.52	[103]
2014	DFS	miR–21 & miR–126	Tissue	103 ccRCC	High risk	19.37	4.060–92.44	[108]
2014	OS	miR–630	Tissue	92 ccRCC	High expression	3.021	2.074–5.726	[97]
2014	DSS	miR–21/miR–10b	Tissue	105 ccRCC	High ratio	2.624	1.201–5.736	[109]
2014	DFS	miR–129–3p	Tissue	48 ccRCC	Low expression	3.119	1.060–9.175	[62]
2014	RFS	miR–125b	Tissue	200 ccRCC	High expression	3.931	1.213–12.74	[110]
2015	OS	miR–497	Tissue	86 ccRCC	Low expression	2.583	1.691–6.361	[111]
2015	DFS	miR–210	Tissue	258 ccRCC	Positive expression	1.910	1.010–3.310	[98]
2015	DFS	miR–126	Tissue	260 ccRCC	Negative expression	0.300 *	0.180–0.500 *	[112]
2015	OS	miR–506	Tissue	106 ccRCC	Low expression	3.886	2.179–7.524	[113]
2015	OS	miR–203	Tissue	90 ccRCC	Low expression	3.071	1.719–6.374	[114]
2015	CSS	miR–21	Tissue	45 RCC	High expression	6.460	1.350–30.94	[115]
2015	CSS	piR–43607	Tissue	68 ccRCC	High expression	1.240 *	1.082–1.445 *	[52]
2015	OS	miR–124–3p	Tissue	62 ccRCC	Low expression	2.600 *	1.069–7.262 *	[116]
2015	RFS	piR–38756	Tissue	72 ccRCC	High expression	3.150	1.960–9.320	[51]
2015	PFS	miR–27a–3p	Tissue	140 ccRCC	High expression	2.710	1.230–6.420	[102]
2016	DFS	miR–194	Tissue	234 ccRCC	Negative expression	0.520	0.270–0.980	[117]
2016	DFS	miR–19a	Tissue	197 ccRCC	High expression	2.410	1.217–4.773	[118]
2016	PFS	miR–222–3p	Tissue	74 ccRCC	High expression	2.020	1.510–2.710	[119]
2017	CSS	miR–223–3p	Tissue	78 ccRCC	High expression	3.510	1.600–7.690	[66]
2017	DFS	miR–10b	Tissue	246 ccRCC	Negative expression	0.470 *	0.280–0.790 *	[120]
2017	OS	miR–766–3p	Tissue	75 RCC	Low expression	2.700	1.310–5.530	[121]
2018	OS	miR–566	Tissue	42 ccRCC	High expression	0.060	0.005–0.769	[122]
2018	OS	miR–18–5p	Tissue	42 RCC	High expression	0.175	0.032–0.953	[123]
2018	OS	miR–663a	Tissue	42 ccRCC	High expression	5.132	1.039–25.350	[124]
2018	OS	miR–572	Tissue	42 RCC	High expression	0.174	0.034–0.878	[125]
2018	RFS	miR–155–5p & miR–210–3p	Tissue	205 ccRCC	High risk	2.700	1.280–5.680	[100]
2018	OS	miR–452–5p	Tissue	102 RCC	High expression	1.580	1.070–2.310	[74]
2019	OS	miR–23a–5p	Tissue	118 RCC	High expression	3.270	1.552–6.893	[126]
2019	OS	miR–183–5p	Tissue	284 ccRCC	High expression	0.550	0.364–0.832	[127]
2019	OS	miR–3133	Tissue	135 ccRCC	Low expression	2.802	1.391–5.646	[128]
2019	OS	miR–221–5p	Tissue	196 ccRCC	High expression	0.550	0.326–0.926	[129]
2019	PFS	piR–51810	Tissue	118 ccRCC	Low expression	0.431	0.190–0.975	[75]
2019	OS	miR–142–3p	Tissue	284 RCC	High expression	0.525	0.347–0.796	[130]
2019	OS	miR–106b–5p	Tissue	284 ccRCC	High expression	0.496	0.327–0.752	[131]
2019	MFS	4 miR panel	Tissue	85 ccRCC	High risk	12.402	3.586–42.893	[132]
2020	DFS	miR–30a–5p^me^	Tissue	227 ccRCC	High methylation	5.174	1.228–21.808	[95]

*—univariable analysis; HR—hazard ratio; 95% CI—95% confidence interval; OS—overall survival; DFS—disease-free survival; PFS—progression-free survival; MFS—metastasis-free survival; CSS—cancer-specific survival; RFS—recurrence-free survival.

**Table 4:** Prognostic small non-coding RNAs (sncRNAs) for RCC in liquid biopsies.

Year	Prognostic Variable	Prognostic Biomarker	Biological Source	Number of Cases	Poor Prognosis	Prognostic Performance		Reference
						HR	95% CI	
2014	CSS	miR–221	Plasma	43 RCC	High expression	10.70	1.330–85.65	[86]
2017	OS	let–7i–5p	Plasma exosomal	65 RCC	Low expression	0.566	0.374–0.857	[133]
2017	CSS	miR–210 & miR–221 & miR–1233	Plasma	50 ccRCC	High risk	3.890	1.260–12.01	[99]
2017	PFS	miR–224	Plasma exosomal	108 ccRCC	High expression	11.00	3.300–68.70	[134]
2017	DSS	miR–150	Plasma	94 ccRCC	Low expression	1.280	1.020–1.670	[91]
2018	PFS	miR–206	Serum	67 ccRCC	High expression	3.670	1.290–10.51	[135]
2020	OS	miR–328–3p	Urine	44 ccRCC	Low expression	0.290	0.080–1.030	[94]
2020	DSS	miR–30a–5p^me^	Urine	53 ccRCC	High methylation	9.376	1.158–75.903	[95]

HR—hazard ratio; 95% CI—95% confidence interval; OS—overall survival; DSS—disease-specific survival; PFS—progression-free survival; CSS—cancer-specific survival.

**Table 5:** Therapy predictive sncRNAs for RCC in tissue, liquid biopsies, and in vitro studies.

Year	Predictive Biomarker	Biological Source	Number of Cases/Cell Lines	Type of Therapy	Main Findings	Reference
2012	multi–miR panels	Whole blood	38 ccRCC	Targeted therapy	Several miRs ⇒ prolonged or poor response to sunitinib	[146]
2013	miR–141	Tissue/in vitro	20 ccRCC	Targeted Therapy	↓ miR–141 ⇒ poor response to sunitinib	[143]
2013	miR–381	In vitro	786–O	Chemotherapy	MiR–381 + 5–FU ⇒ lower proliferation, and ↑ 5–FU efficacy	[147]
2014	miR–942	Tissue/in vitro	20 RCC & Caki–2	Targeted Therapy	MiR–942 ⇒ sunitinib resistance ⇒ ↓ TTP & OS	[148]
2014	miR–200c	In vitro	HEK293, SN12C, ACHN, 786–O & Caki–1	Targeted Therapy	Mimic miR–200c ⇒ sensitivity to therapy with TKI	[149]
2015	miR–27b	In vitro/in vivo	ACHN, 769–P, 786–O & Caki–1	Chemotherapy	Overexpressing miR–27b ⇒ sensitizes RCC cells to a variety of anti–cancer drugs, such as doxorubicin	[150]
2015	miR–30a	Tissue/in vivo	10 ccRCC & A498 + 786–O	Targeted Therapy	Exogenously expression of miR–30a ⇒ ↑ sorafenib treatment efficacy	[151]
2015	miR–200c	In vitro	Caki–1, Caki–2, A498, ACHN, 786–O & 769–P	Chemotherapy	↓ miR–200c ⇒ resistance to docetaxel	[152]
2015	miR–155 & miR–484	Tissue	63 RCC	Targeted Therapy	↓ of both miRs ⇒ better response to sunitinib ⇒ ↑ TTP	[144]
2015	miR–124	In vitro	Caki–2	Chemotherapy	↓ miR–124 ⇒ resistance to doxorubicin and vinblastine	[153]
2015	miR–221 & miR–222	Tissue/in vivo	30 ccRCC & 786–O + ACHN	Targeted Therapy	↑ of both miRs ⇒ poor response to sunitinib therapy	[154]
2016	miR–99b–5p	Tissue	40 ccRCC	Targeted Therapy	↓ miR–99b–5p ⇒ ↓ PFS and in TKI non–responders	[155]
2017	miR–605	Serum	36 ccRCC	Targeted Therapy	MiR–605 ⇒ ↓ after vorinostato and bevacizumab therapy in responders	[156]
2017	miR–27b & miR–23b & miR–628–5p	Tissue	123 RCC	Targeted Therapy	↑ of these miRs ⇒ long–term sunitinib response	[157]
2017	miR–144–3p	In vitro/in vivo	786–O & SN12–PM6 + Nude mice	Targeted Therapy	↑ miR–144–3p ⇒ ↓ ARID1A and resistance to sunitinib	[158]
2017	miR–451	In vitro	ACHN & GRC–1	Chemotherapy	MiR–451 knockdown ⇒ ↑ sensitivity to adriamycin therapy	[159]
2018	miR–942 & miR–133	Tissue	56 RCC	Targeted Therapy	Both miRs ⇒ discriminate between sunitinib responders and non–responders	[160]
2019	miR–421	Tissue	101 MRCC	Targeted Therapy	↑ miR–421 in TKI non–responders	[145]
2019	miR–376b–3p	Tissue	132 ccRCC	Targeted Therapy	↓ miR–376b–3p in sunitinib poor responders	[161]
2020	miR–31–5p	Exosomes from plasma/in vitro/in vivo	40 PD MRCC + 786–O + BALB/c nude mice	Targeted Therapy	↑ miR–31–5p in PD vs non–PD patients’ plasma samples treated with sorafenib	[162]

RCC—renal cell carcinoma; ccRCC—clear cell renal cell carcinoma; 5–FU—5–fluorouracil; TTP—time to progression; OS—overall survival; PFS—progression-free survival; TKI—tyrosine kinase inhibitors; mRCC—metastatic renal cell carcinoma; PD—progressive disease; non-PD—non-progressive disease; ↓—downregulation; ↑—upregulation.

**Table 6:** Long non-coding RNAs (LncRNAs) as potential diagnostic biomarkers in RCC tissues and liquid biopsies.

Year	Diagnostic Biomarker	Biological Source	Number of Cases/Controls	Diagnostic Performance			Reference
				Sensitivity (%)	Specificity (%)	AUC	
1999	aHIF	Tissue	10 ccRCC; 7 pRCC	n.a.	n.a.	n.a.	[175]
2011	aHIF	Tissue	26 RCC; 26 MNT	n.a.	n.a.	n.a.	[176]
2014	AK096725	Tissue	70 RCC; 70 MNT	n.a.	n.a.	n.a.	[179]
2015	TTC34–3	Tissue	55 ccRCC; 52 NRT	n.a.	n.a.	0.990	[180]
2015	CYP4A22–2/3	Tissue	102 ccRCC; 50 NRT	n.a.	n.a.	0.790	[177]
2016	TRIM52–AS1	Tissue	60 RCC; 60 MNT	n.a.	n.a.	n.a.	[181]
2016	UCA1	Tissue	46 RCC; 46 MNT	n.a.	n.a.	n.a.	[182]
2016	UC009YBY.1	Tissue	70 RCC; 70 MNT	54.3	82.9	0.700	[178]
2018	HOTAIR	Tissue	24 ccRCC; 24 MNT	n.a.	n.a.	0.923	[70]
2016	5 lncRNA panel	Serum	24 ccRCC; 27 AC	79.2	88.9	0.900	[183]
2018	GIHCG	Serum	31 Stage I ccRCC; 46 AC	80.7	84.8	0.886	[184]

MNT—matched normal tissue; NRT—renal normal tissue; RCC—renal cell carcinoma; ccRCC—clear cell renal cell carcinoma; pRCC—papillary renal cell carcinoma; AC—asymptomatic controls; n.a.—not available.

**Table 7:** LncRNAs as potential prognostic biomarkers in RCC tissues.

Year	Prognostic Variable	Prognostic Biomarker	Biological Source	Number of Cases	Poor Prognosis	Prognostic Performance		Reference
						HR	95% CI	
2014	OS	CADM1–AS1	Tissue	64 ccRCC	Low expression	0.211	0.088–0.504	[188]
2014	OS	SPRY4–IT1	Tissue	98 ccRCC	High expression	3.375	1.824–7.391	[189]
2015	OS	NBAT–1	Tissue	98 ccRCC	Low expression	3.701	1.261–9.784	[190]
2015	OS	MALAT1	Tissue	106 ccRCC	High expression	3.086	1.813–7.025	[191]
2015	OS	H19	Tissue	92 ccRCC	High expression	3.894	1.872–8.014	[192]
2015	PFS	ZNF180–2	Tissue	102 ccRCC	Low expression	0.803	0.699–0.922	[177]
2016	OS	Linc00152	Tissue	77 ccRCC	High expression	2.577	1.233–5.387	[193]
2016	RFS	lncARSR	Tissue	205 ccRCC	High expression	2.023	1.213–3.375	[185]
2017	OS	TUG1	Tissue	203 ccRCC	High expression	2.337	1.451–6.673	[194]
2017	OS	TCL6	Tissue	71 ccRCC	Low expression	0.130	0.020–0.680	[195]
2017	OS	SLINKY	Tissue	100 ccRCC	High expression	8.440	1.770–40.23	[196]
2017	OS	PANDAR	Tissue	62 ccRCC	High expression	1.130	0.980–5.120	[197]
2017	OS	MRCCAT1	Tissue	68 ccRCC	High expression	2.306	1.003–2.849	[198]
2017	OS	PVT1	Tissue	50 ccRCC	High expression	1.494	1.081–2.063	[199]
2017	DFS	MFI2–AS1	Tissue	167 ccRCC	Positive expression	4.240	2.070–8.700	[200]
2017	DFS	PVT1	Tissue	129 ccRCC	High expression	3.553	1.515–8.329	[186]
2018	OS	ENSG00000241684	Tissue	61 ccRCC	Low expression	5.378	2.084–13.88	[201]
2018	OS	LUCAT1	Tissue	64 ccRCC	High expression	3.650	1.356–9.826	[202]
2018	OS	CRNDE	Tissue	112 ccRCC	High expression	2.023	1.039–3.468	[203]
2019	OS	EGFR–AS1	Tissue	204 RCC	High expression	2.204	1.145–4.241	[187]
2016	PFS	lncARSR	Plasma	71 ccRCC	High expression	n.a.	n.a.	[204]
2018	OS	GIHCG	Serum	46 ccRCC	High expression	n.a.	n.a.	[184]

HR—hazard ratio; 95% CI—95% confidence interval; OS—overall survival; DFS—disease-free survival; PFS—progression-free survival; RFS—recurrence-free survival; RCC—renal cell carcinoma; ccRCC—clear cell renal cell carcinoma; n.a.—not available.

**Table 8:** Therapy-predictive lncRNAs for RCC in tissue, liquid biopsies, and in vitro studies.

Year	Predictive Biomarker	Biological Source	Number of Cases/Cell Lines	Type of Therapy	Main Findings	Reference
2016	lncARSR	Plasma	71 ccRCC	TargetedTherapy	↑ lncARSR in progressive disease during sunitinib therapy	[204]
2017	lncSRLR	Tissue/in vitro	51 RCC; RCC cell lines	TargetedTherapy	↑ lncSRLR in sorafenib non–responders. In vitro ablation ⇒ ↑ therapeutic sensitivity	[205]
2017	SARCC	In vitro	RCC cell lines	TargetedTherapy	↑ SARCC after sunitinib treatment ⇒ ↓ sunitinib resistance (positive feedback loop)	[206]
2019	GAS5	Tissue/ in vitro/ in vivo	15 ccRCC; RCC cell lines; mice model	TargetedTherapy	↓ GAS5 in sorafenib non–responders; in vitro/ in vivo re–introduction ⇒ ↑ sensitivity	[207]

RCC—renal cell carcinoma; ccRCC—clear cell renal cell carcinoma; ↑—upregulated; ↓—downregulation.
